# Reserpine-induced fibromyalgia model in female rats is a long-lasting painful condition masked by antinociceptive endogenous opioids

**DOI:** 10.3389/fphar.2026.1733691

**Published:** 2026-03-18

**Authors:** Cristina Juárez-Núñez, Pedro Segura-Chama, Juan Miguel Pizaña-Encarnación, Francisco Pellicer, Vinicio Granados-Soto, Francisco Mercado, Angélica Almanza

**Affiliations:** 1 Laboratorio de Fisiología Celular, Instituto Nacional de Psiquiatría Ramón de la Fuente Muñiz, Ciudad de México, Mexico; 2 Investigador por México, Secretaría de Ciencias, Humanidades, Tecnología e Innovación, Ciudad de México, Mexico; 3 Neurobiology of Pain Laboratory, Departamento de Farmacobiología, Cinvestav, South Campus, Mexico City, Mexico; 4 Laboratorio de Neurofisiología Integrativa, Instituto Nacional de Psiquiatría Ramón de la Fuente Muñiz, Ciudad de México, Mexico

**Keywords:** fibromyaglia, opioid, opioid receptor, pain, reserpine

## Abstract

**Background:**

Fibromyalgia (FM) is a chronic painful condition that primarily affects women. The allodynia and hyperalgesia induced by reserpine is a FM model commonly used to study the disease; however, it produces one painful episode, in contrast to FM, which is a chronic painful condition. Reserpine induces hypersensitivity for 10 days, which is resolved by day-21. In this study, we sought to determine whether the resolution of pain was due to the release of endogenous antinociceptive opioids and the activation of spinal opioid receptors.

**Methods:**

Female Wistar rats were used in this study. To induce a fibromyalgia-like model, reserpine was subcutaneously administered. Twenty-one days after reserpine administration, subcutaneous or intrathecal opioid blockers were administered to evaluate latent sensitization. Additionally, serum β-endorphin concentration was determined by ELISA.

**Results:**

Reserpine produced transitory hyperalgesia and allodynia, which was resolved by day 21. Systemic administration of naloxone on days 21, 33, and 45 reinstated hypersensitivity. Likewise, intrathecal administration of naltrexone or specific antagonists of **μ** and **δ** opioid receptors (CTOP and naltrindole) reinstated hypersensitivity. These results suggest that an increased concentration of opioid agonists in reserpinized animals leads to antinociceptive effects. Accordingly, reserpine injection enhanced serum β-endorphin concentration in female rats.

**Conclusion:**

Our data suggest that reserpine-induced nociceptive hypersensitivity is a long-lasting condition masked by the compensatory release of β-endorphin and the activation of spinal µ and, to a lesser extent, δ opioid receptors.

## 1 Introduction

Fibromyalgia (FM) is a clinically defined syndrome characterized by widespread chronic pain, sleep problems and fatigue, with an estimated incidence between 2% and 4% of the general population (Wolfe et al., 2010; Queiroz, 2013; Sarzi-Puttini et al., 2020). However, the exact cause of FM remains unclear. There are reports in which lower concentrations of monoaminergic neurotransmitter metabolites were found in the cerebrospinal fluid of patients with FM than in healthy controls, and this imbalance is postulated as a possible cause of hyperalgesia in these patients (Russell et al., 1992). In addition, sympathetic ganglia dysfunction, immune response against glial cells in the dorsal root ganglion (DRG), and neutrophil infiltration have been proposed as key factors in the development of sensory sensitization and subsequent pain (Goebel et al., 2021; Martínez-Lavín, 2021; Caxaria et al., 2023). Because FM is a heterogeneous syndrome, multimodal treatment approaches have been suggested (Sluka and Clauw, 2016; Wolfe et al., 2016; Sarzi-Puttini et al., 2020).

Several animal models have been developed to study FM (Brum et al., 2022), where nociceptive hypersensitivity is triggered by stress, hyperalgesic priming, or reserpine (Nagakura et al., 2009). The latter resembles many features of the disease: generalized hyperalgesia and allodynia refractory to opioids and non-steroidal anti-inflammatory drugs (NSAID), while serotonin and norepinephrine reuptake inhibitors (SNRIs) have a partial antinociceptive effect (Aster et al., 2022). The reserpine-induced model of FM also recapitulates one of the proposed causes of the disease: a decrement in monoaminergic neurotransmission (Russell et al., 1992; Nagakura et al., 2009). The painful condition in the reserpine model is transitory, which contrast with the chronic course of the pain in FM patients (Nagakura et al., 2009).

The origin of the painful condition and the mechanisms by which it is maintained in this model are not fully understood; however, microglial activation, acid-sensing ion channel 3 overexpression, D1-like dopamine receptor activation, and overexpression and activation of α_5_ subunit-containing GABA_A_ receptors are some of the mechanisms found in this model (Taguchi et al., 2015; De la Luz-Cuellar et al., 2019; 2023).

In inflammatory and some models of neuropathic pain, allodynia and hyperalgesia resolve after a few weeks of insult. Under these conditions, the administration of naloxone (an opioid receptor antagonist) precipitates nociceptive hypersensitivity, suggesting that allodynia is masked by endogenous opioids. This phenomenon is called latent sensitization (Corder et al., 2013; Marvizon et al., 2015; Walwyn et al., 2016; Severino et al., 2018; Inyang et al., 2021; Morales-Medina et al., 2023).

Because the resolution of hypersensitivity in the reserpine model occurs in a short period, in this study, we sought to determine whether opioid-induced latent sensitization occurs in the reserpine model, and if masks the allodynia and hyperalgesia.

## 2 Materials and methods

### 2.1 Animals

A total of 69 Female Wistar rats were used in this study (200–330 g). Animals were housed in acrylic cages with wood chip beds and soft enrichment in a climatized room (22 °C ± 2 °C), with *ad libitum* access to food (rat chow) and water before and during the experiments, and 12 h light/dark cycles. At the end of the experimental protocol all the animals were euthanized in a CO_2_ chamber (at 99.9%). All procedures followed the Guide of Care and Use of Laboratory Animals of the National Academy of Science of the USA, local regulations (NOM-062-ZOO-1999) and the recommendations of the Guidelines on Ethical Standards for Investigation of Experimental Pain in Animals (Zimmermann, 1983). The study protocol was approved by the Institutional Laboratory Animal Care Committee (approval folio CICUAL/04/2024) and ethics committee (CEI/C/032/2025).

### 2.2 Drugs

Reserpine (Cat. sc-203370), naltrindole (Cat. sc-202236), naloxone (Cat. sc-203153A) and naltrexone (Cat. sc-477813) were obtained from Santa Cruz Biotechnology (Dallas, TX, USA). D-Pen-Cys-Tyr-D-Trp-Orn-Thr-Pen-Thr-NH_2_ (CTOP; Cat. P5296) and nor-Binaltorphimine (nor-Bin; Cat. N1771) were obtained from Sigma-Aldrich (St. Louis, MO, USA). Reserpine was dissolved in glacial acetic acid (at a final concentration of 0.5%), naltrindole was dissolved in ethanol (at a final concentration of 1%), and the remaining drugs were dissolved in sterile saline (NaCl 0.9%). Doses and concentrations were obtained from previous reports (Back et al., 2006; Nagakura et al., 2009; Ju et al., 2013; Marvizon et al., 2015; Custodio-Patsey et al., 2020) or determined in pilot experiments under our conditions.

### 2.3 Drug administration

A 27G or 31G needle was used for subcutaneous (s.c.) administration. The animal’s head and forelimbs were covered with a soft blanket near the neck, the skin was gently stretched, and the injection was applied to skin that was not attached to the animal’s back, the final volume injected was 1 mL/kg. For intrathecal (i.t.) injection, drug administration was performed as previously described (De La Calle and Paíno, 2002; Almanza et al., 2015). Briefly, animals were anesthetized with isoflurane (2%), a puncture at L4-L5 or L5-L6 was administered with a 27G needle, and 50 µL of the solution (vehicle or drug) was delivered.

### 2.4 Induction of the reserpine model

To induce the reserpine model, reserpine was subcutaneously injected (1 mg/kg) over the neck every 24 h for three consecutive days. The control groups received 0.5% acetic acid in saline (1 mL/kg, s.c.) (Nagakura et al., 2009).

### 2.5 Behavioral assays

Animals were habituated to each behavioral apparatus for 30 min on three consecutive days before carrying out the experiment. The flexor reflexes were tested using both thermal and mechanical stimuli. The thermal stimulus was carried out using a Hargreaves apparatus (Mod. 37450, Ugo Basile, Varese, Italy), in which an infrared lamp (50 W) was placed under the hind paw of the animal through an anti-reflective glass, the latency of the flexor reflex was measured automatically (lamp power was established at 40% to produce a flexor reflex in approximately 10 s). An average of three measurements in each paw was considered as the latency at a given moment; at least 1 minute was left between measurements, a cutoff of 25 s was established to avoid any tissue damage.

Mechanical stimuli were applied using a dynamic plantar aesthesiometer (Mod. 37370, Ugo Basile, Varese, Italy). A metallic filament was used as a probe and placed below the hind paw of the animal, resting on a wire mesh. A stimulus with an increasing force from 0 to 50 g was used (ramp duration was 10 s, and the increase in force was linear over time). Two values directly correlated with each other were obtained from the experiments: latency and force, both of which were automatically recorded by the apparatus. The average of four measurements in each paw was considered the threshold at any given moment, and at least 1 minute was left between measurements, a cutoff of 25 s was established to avoid any tissue damage.

Baseline measurements in both tests were taken before reserpine administration and at 2, 5, 7, 10, 15, and 21 days after reserpine administration. In some experiments, latencies were recorded on days 33 and 45. During the experiments the same cohort of animals were evaluated the time indicated for each case (most of the experiments last 21 days after reserpine or vehicle administration). When a drug was tested after the resolution of the reserpine effect on the nociceptive response to thermal and mechanical stimuli, baseline of the test day (usually on day 21) was taken and the drug effect (subcutaneous or intrathecal administration), was evaluated by measuring the latencies or the threshold at 15, 30, 60, and 120 min after drug administration (s.c. or i.t.).

### 2.6 Ovariectomy

Bilateral ovariectomy was performed as previously described (Hernandez-Leon et al., 2018). Under isoflurane anesthesia (2.5% for induction and 2% for maintenance), skin hair was fully trim, and skin was cleaned with povidone-iodine solution at 10%. Ovaries were surgically removed from the lower central abdominal cavity. After surgery, the animals were administered with s.c. meloxicam for 3 days (0.5 mg/kg once a day) and were allowed to recover for a minimum of 15 days. On that day, the presence of the diestrus phase of the estrous cycle was confirmed using a vaginal smear.

### 2.7 Quantification of rat β-endorphin plasma concentration

Serum samples were obtained under isoflurane anesthesia during the light phase (09:00–12:00) to minimize circadian variations in β-endorphin levels. Animals were anesthetized with isoflurane (2%–3% in oxygen) for 3–5 min prior to blood collection to minimize stress-induced β-endorphin release. Three milliliters of blood was extracted from each rat’s heart using cardiac puncture. Blood samples were immediately collected in serum separator tubes (BD Vacutainer SST Tube Plastic Gold 3 mL) and centrifuged at 2000 × *g* for 15 min at room temperature to separate the serum from the cellular components. The resulting serum was stored at −80 °C until further analysis, which was performed within 30 days of collection (Pizaña-Encarnación et al., 2025).

Serum β-endorphin levels in rats were determined by enzyme-linked immunosorbent assay according to the manufacturer’s instructions (Rat Beta-Endorphin ELISA Kit, Cat. MBS763627, MyBioSource, San Diego, CA). Serum β-endorphin levels were assayed in duplicate, and all values were expressed as the mean ± standard error of the two measurements.

### 2.8 Statistical analysis

Pharmacological experiments were performed by an experimenter blinded to the treatments. The number of animals in each experimental group was set to six, and the sample size was calculated using G-power software (Faul et al., 2007), establishing an α of 0.05, and a 1-β value of 0.8. The differences in the means and dispersion of data between the groups were estimated from the pilot experiments. Pharmacological administration, behavioral analyses, and randomization were performed by the same experimenter to minimize potential confounders. The animals were randomly assigned to groups by simple randomization.

Behavioral data were normalized as change percentage, which is the obtained value under the effect of the vehicle or a drug (at any time point) divided by the mean baseline value before reserpine administration and are presented as the mean ± SEM of six animals per group. The area under the curve (AUC) was calculated using the trapezoid method, considering the percentage change over time for each drug and concentration.

Statistical differences between groups were determined by the statistical analysis indicated in each figure legend. All statistical analyses and plots were performed using GraphPad Prism 8 or 10 (GraphPad Software Inc., La Jolla, CA, USA). *P* values less than 0.05 (*P* < 0.05) were considered significant, a complete description of the statistical results are presented in Supplementary Table S1.

## 3 Results

### 3.1 Reserpine induced transitory hypersensitivity in nociceptive tests which is reinstated by naloxone

**FIGURE 1:**
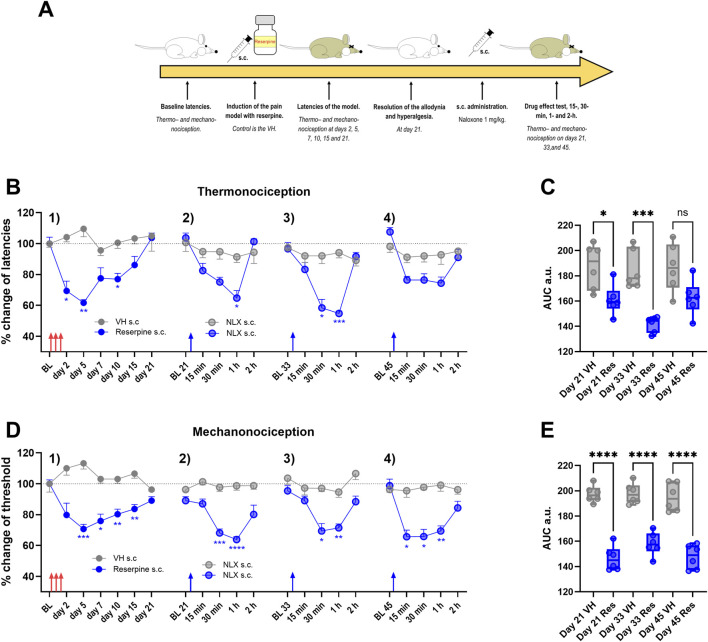
Latent sensitization phenomena in the reserpine-model. In **(A)** the experimental design is illustrated. In **(B)**, the reserpine or vehicle s.c. application is shown as percentage of change of latencies, in 1) after reserpine administration hypersensitivity to thermonociceptive stimulus is developed, but not with the VH. The hypersensitivity improved until total recovery by day 21. In 2) on day 21, naloxone (NLX) s.c. application produced a transitory recall of the hypersensitivity in reserpinized animals but not in the controls, similarly happened on day 33 and 45 (subsection 3) and 4), respectively). In **(C)**, area under the curve (AUC) analysis obtained from data in **(B)** at the different days. In **(D)**, similarly to **(B)**, but upon mechanonociceptive test showed as change percentage. In **(E)**, AUC analysis obtained from data in **(D)** * *P* < 0.05, ** *P* < 0.01, *** *P* < 0.001 and **** *P* < 0.0001 comparing NLX effect between VH and reserpine treated animals (two-way ANOVA with Sidák’s *post hoc* test in **(B,D)**; one-way ANOVA with Tukey’s *post hoc* test in **(C,E)**. BL, baseline; s.c., subcutaneous. Red arrows indicate reserpine or VH administration, and blue arrows the naloxone s.c. administration.

The experimental design of the following results is illustrated in Figure 1A. Reserpine, but not vehicle administration reduced the thermal latency and withdrawal threshold in female rats in a time-dependent manner, which was interpreted as thermal hyperalgesia (thermonociception) and mechanical allodynia (mechanonociception; subsection 1 in Figures 1B,D). The maximal effect of reserpine was observed on day 5 after the last reserpine injection in both tests. The response to thermonociceptive and mechanociceptive stimuli returned to baseline values (remission) by day 21 after reserpine injection (Subsection 1, in Figures 1B,D).

Because remission of inflammatory or neuropathic pain could be due to tonic inhibition mediated by opioid receptors, we evaluated whether latent nociceptive sensitization occurred in the reserpine model. We found that naloxone injections (1 mg/kg, s.c., given at 21, 33, and 45 days after reserpine administration) induced transient hypersensitivity in thermonociception (Subsections 2, 3, and 4; Figure 1B) and mechanonociception (Subsections 2, 3, and 4; Figure 1D) in reserpinized animals, but not in controls. These observations were corroborated in the AUC analysis, where naloxone s.c. effect in non-reserpinized animals was significantly different from the naloxone effect in reserpinized animals (Figures 1C,E).

### 3.2 Reserpine induced transitory hyperalgesia and allodynia which were reinstated by intrathecal naltrexone

**FIGURE 2:**
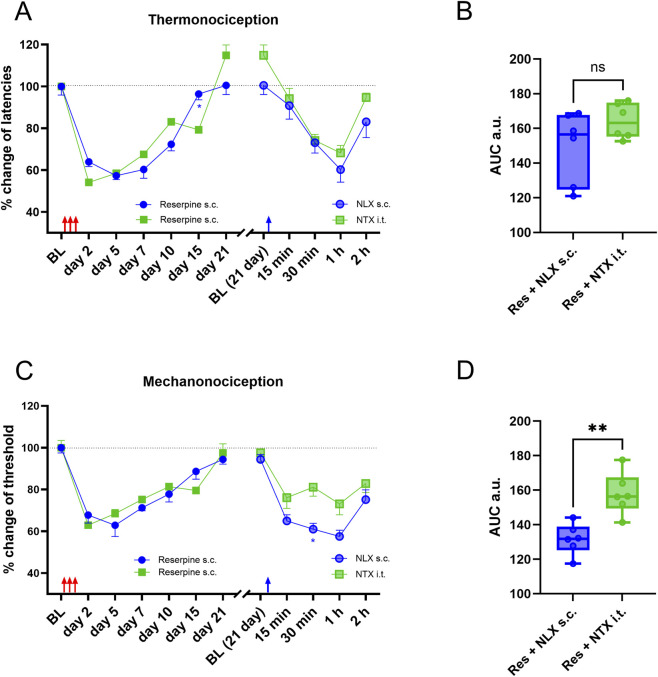
Subcutaneous naloxone had a similar effect to intrathecal naltrexone (NTX), in restoring hyperalgesia and allodynia. In **(A)**, it is shown the latencies as change percentage upon thermonociceptive test of two groups of female rats, animals were reserpinized and left to recover from hyperalgesia for 21 days, then s.c. naloxone or i.t. naltrexone was applied, there were no significant differences in the recall of hyperalgesia. In **(B)**, AUC analysis obtained from data in **(A)**, right. In **(C)**, similarly as in **(A)**, but upon mechanonociceptive test. In **(D)**, AUC analysis obtained from data in **(C)**, right. Red and blue arrows indicate reserpine and NTX administration, respectively. * *P* < 0.05 and ** *P* < 0.01 compared between groups (two-way ANOVA with Sidák’s *post hoc* test in **(A,C)**; one-way ANOVA with Tukey’s *post hoc* test in **(B,C)**. BL, baseline; s.c., subcutaneous, i.t., intrathecal. Red arrows indicate reserpine or VH administration, and blue arrows the naloxone s.c. administration or naltrexone i.t. administration.

To test whether spinal opioid receptors are responsible for latent sensitization, we compared the effects of subcutaneous naloxone in rats previously treated with reserpine with intrathecal injection of naltrexone (100 µg), whose potency and efficacy is similar to those of naloxone (Sirohi et al., 2009). I.t. naltrexone induced transient hypersensitivity in thermonociception and mechanonociception, similar to that induced by systemic naloxone (Figures 2A,C). AUC analysis confirmed a significant reduction in thermal latencies in the reserpinized animals after intrathecal administration of naltrexone, with no differences from systemic naloxone (Figure 2B). In the mechanical stimuli AUC comparison between systemic naloxone and i.t. naltrexone, naloxone produced a significantly larger effect than i.t. naltrexone (Figure 2D).

### 3.3 Participation of µ, δ and κ opioid receptors in the latent state of sensitization in the reserpine model

**FIGURE 3:**
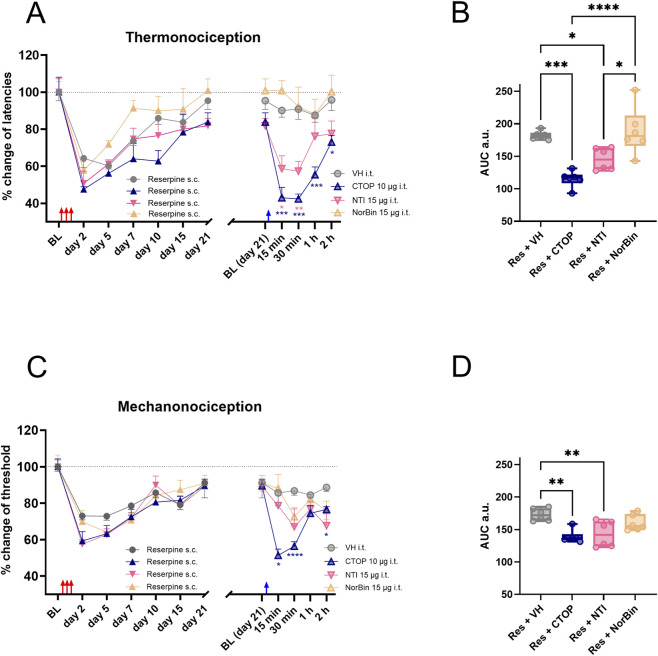
Effect of μ, δ and κ opioid receptor specific antagonists, on latent sensitization, the experimental design is the same as in Figure 1A. In **(A)**, the results of thermonociceptive tests as percentage of change are shown. On day 21, when the latencies of reserpinized animals were close to baseline (BL), μ opioid-receptor antagonist CTOP, δ opioid-receptor antagonist naltrindole (NTI), κ opioid-receptor antagonist norbinaltorphimine (NorBin) or vehicle (VH) were i.t. applied in four groups separately. In **(B)**, AUC analysis obtained from data in **(A)**, right. In **(C)**, like **(A)**, but in the mechanonociceptive test, showed as percentage of change. In **(D)**, AUC analysis obtained from data in **(C)**, right. * *P* < 0.05, ** *P* < 0.01, *** *P* < 0.001 and **** *P* < 0.0001 comparing the different opioid antagonists i.t. effect with VH in reserpinized animals (two-way ANOVA with Sidák’s *post hoc* test in **(A,C)**; one-way ANOVA with Tukey’s *post hoc* test in **(B,C)**. Red arrows indicate reserpine administration and blue arrows VH or opioid-receptors antagonist administration.

To determine the participation of spinal µ opioid receptors in the latent sensitization of the reserpine model, intrathecal CTOP (selective µ opioid receptor antagonist), naltrindole (selective δ opioid receptor antagonist) and nor-Bin (selective κ opioid receptor antagonist) were administered in reserpinized rats on day 21. CTOP (10 µg), but not the vehicle, fully reinstated hypersensitivity in thermonociception (Figures 3A,B), and mechanonociception (Figures 3C,D) in reserpine treated female rats. The AUC analysis confirmed the reduction in thermal latency and withdrawal threshold induced by CTOP compared to the vehicle (Figures 3B,D).

I.t. naltrindole (15 µg), but not vehicle, induced transient hypersensitivity in thermonociception and, to a lesser extent, in mechanonociception in reserpinized animals (Figures 3A,C). The AUC analysis confirmed the reduction in thermal latency and withdrawal threshold induced by naltrindole compared to the vehicle (Figures 3B,D). In contrast, i.t. nor-Bin (15 µg) did not induce hypersensitivity to thermonociception nor mechanonociception (Figures 3A,C). The AUC analysis confirmed the lack of the nor-Bin effect, which was indistinguishable from vehicle administration (Figures 3B,D).

### 3.4 The recall of thermonociception and mechanonociception by blockade of opioid receptors is independent on female gonadal hormones

Because our study focuses on female rats, we investigated the role of ovarian hormones in latent sensitization induced by reserpine. For these experiments, subcutaneous naloxone was used to show the latent state of sensitization owing to the convenience of applying the opioid antagonist *via* the systemic route.

**FIGURE 4:**
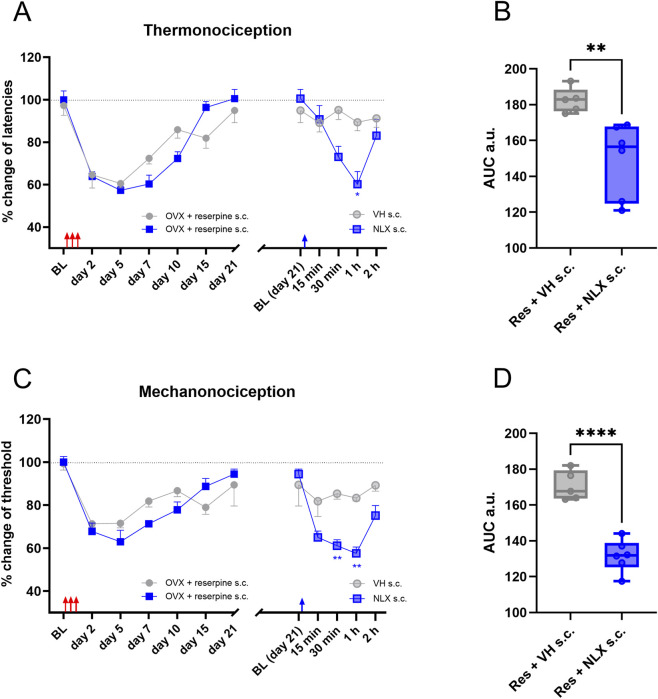
The ovariectomy (OVX) did not modify the recall of hyperalgesia and allodynia produced by opioid antagonists. In **(A)**, the latencies in response to thermonociceptive test are shown as change percentage. After reserpine administration (red arrows), animals were left for recovery for 21 days, s.c. NLX produced a recall of the hyperalgesia in the reserpine treated group, but the s.c. administration of the VH produced no change in the latencies. In **(B)**, AUC analysis obtained from data in **(A)**, right. In **(C)**, similarly to **(A)**, but upon mechanonociceptive test showed as percentage of change. In **(D)**, AUC analysis obtained from data in **(C)**, right. * *P* < 0.05, ** *P* < 0.01 and **** *P* < 0.0001 comparing s.c. NLX effect with VH in reserpinized animals (two-way ANOVA with Sidák’s *post hoc* test in **(A,C)**; one-way ANOVA with Tukey’s *post hoc* test in **(B,C)**. Red and blue arrows indicate reserpine and NLX administration respectively.

Reserpine induced hypersensitivity in thermonociception and mechanonociception in ovariectomized rats in a manner similar to that observed in non-ovariectomized animals. In this case, remission of hypersensitivity was observed 21 days after the reserpine injection. Under these conditions, systemic administration of naloxone (1 mg/kg, s.c.) fully restored hypersensitivity in thermonociception and mechanonociception (Figures 4A,C). AUC analysis confirmed the reduction in thermal latency and withdrawal threshold due to naloxone compared to the vehicle group (Figures 4B,D).

### 3.5 Reserpine treatment increases serum β-endorphin levels in rats

**FIGURE 5:**
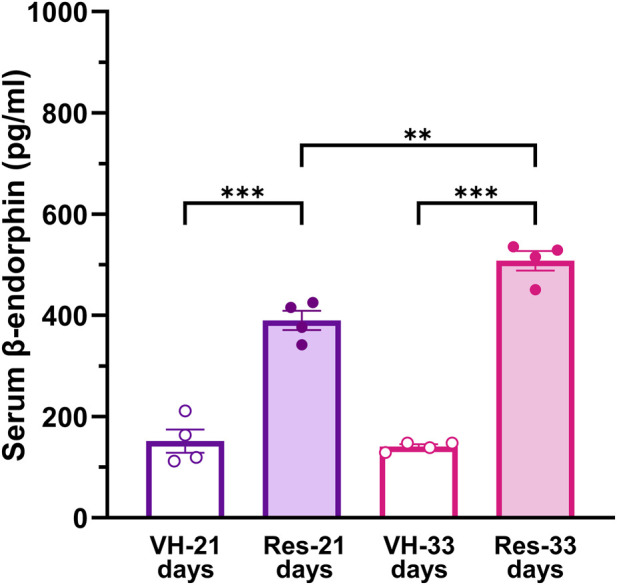
Quantification of β-endorphin in female rats in the reserpine-model. Serum β-endorphin levels (pg/mL) were measured in female rats treated with vehicle (VH) or reserpine (Res) at different time points. Reserpine on day 21produced a significant increase in β-endorphin serum levels compared with VH administration (***P < 0.001, one-way ANOVA with Tukey’s *post hoc* test). A further increase in β-endorphin levels was found on day 33 after reserpine administration, which was different to the VH (**P < 0.001) and the reserpine effect at day 21 (**P < 0.01), indicating a progressive elevation of endogenous opioids plasma concentration over time.

We investigated whether remission of reserpine-induced nociceptive hypersensitivity is associated with enhanced serum β-endorphin levels. Reserpine treatment increased serum β-endorphin levels during the remission process at 21 (*P* < 0.001) and 33 (*P* < 0.001) days after reserpine injection compared to that in vehicle-treated rats (Figure 5).

## 4 Discussion

The present study investigated the nature of the apparent resolution of a pain-like condition induced by reserpine, a commonly used model of FM-like pain (Brum et al., 2022). We found that once hyperalgesia and allodynia were resolved, pharmacological blockade of opioid receptors, either systemic or at the spinal cord level, produced a complete recall of the painful signs. These results suggest that reserpine leads to a compensatory mechanism, in which an increase in endogenous opioid release produces a significant reduction in nociceptive hypersensitivity.

In this study, latent sensitization was revealed in the reserpine model, and it was mediated by opioid receptors. To the best of our knowledge, this is the first report on latent sensitization in a FM model. Our results agree with previous observations in other models of inflammatory and neuropathic pain (Corder et al., 2013; Severino et al., 2018). Our data suggest that activation of opioid receptors at the spinal cord level is sufficient to develop a long-lasting and steady antinociceptive state in animals.

Here, we showed that the activation of spinal µ and, to a lesser extent, δ opioid receptors are necessary for pain remission, while κ opioid receptor participation seems negligible. This statement is based on the following evidence: i) reserpine induces time-dependent allodynia and hyperalgesia, which spontaneously remit in female rats; ii) systemic naloxone and spinal naltrexone reinstated reserpine-induced nociceptive hypersensitivity in female rats with similar efficacy; iii) selective blockade of spinal µ- and δ-opioid receptors induced transient thermonociception and mechanonociception at 21 days in female rats; and iv) reserpine, but not vehicle, enhanced serum β-endorphin levels in female rats (Gomes et al., 2020).

If part of the observed results is due to a change in the expression of opioid receptors is something that requires further research, but it has been suggested that an upregulation of µ-opioid receptor could happened in latent sensitization after CFA-induced inflammatory pain (Corder et al., 2013). Previous studies have suggested that NK1 (Chen and Marvizon, 2020), neuropeptide Y (Fu et al., 2020), and α_2_ adrenergic receptors (Walwyn et al., 2016) participate in latent sensitization after inflammatory and neuropathic pain. Whether these receptors also participate in reserpine-induced latent sensitization remains unknown and requires further investigation.

We observed that restoration of allodynia was possible with a subcutaneous injection of naloxone for 45 days, with no signs of resolution. This long-time frame of hypersensitivity could lead to the study of the effectiveness of pharmacological agents against the syndrome with a long-term follow-up, supervising if naloxone-produced painful-episodes are modified by some treatment, while the animal could be free from constant allodynia between episodes. This tool for studying FM as a model of long-lasting pain could be complementary to those models recently developed, in which repetitive administration of reserpine induces a chronic, non-transitory, painful condition (allodynia) (Álvarez-Pérez et al., 2022) or repeated acidic-saline muscular injection, which is also a long-lasting permanent painful model (Sluka et al., 2001; Brum et al., 2022).

There is controversy regarding β-endorphin concentration changes in patients with FM compared to healthy controls. There are reports where no changes have been found in the serum concentrations (Yunus et al., 1986) or cerebrospinal fluid (Vaerøy et al., 1991). In contrast, a decrease in the concentration of β-endorphins in immune cells has also been reported (Panerai et al., 2002). This discrepancy could be due to the classification method of the patients or sensitivity of the assays. Remarkably, other recent studies have found greater endogenous opioid concentrations in plasma and cerebrospinal fluid, similarly as happened after reserpine administration. Met-enkephalin concentrations in the cerebrospinal fluid of patients with FM are significantly higher than those in healthy controls (Baraniuk et al., 2004). In a recent report, in agreement with our results, β-endorphin plasma concentration was found to be significantly higher in FM patients than in controls, paired with sex, age, and body mass index (Sarzi-Puttini et al., 2020; Navarro-Gonzalez et al., 2025).

These results may explain why most FM patients and animal models are refractory to the antinociceptive effects of opioid receptor agonists (Sluka et al., 2001; Nagakura et al., 2009; Sarzi-Puttini et al., 2020). Tramadol is prescribed for the treatment of FM; however, it is useful for pain management in only a small fraction of patients (Littlejohn et al., 2016; Dailey et al., 2022). This lack of efficacy could be the result of high β-endorphin plasma levels, which could completely occupy their receptors, resulting in ineffective opioid receptor agonist treatment. Low doses of NTX (6 mg) have been used for the treatment of FM (Johnson et al., 2014), based in the assumption that there is an imbalance in the opioidergic system in FM, whose final result is pain in the patients. However, several reports coincide in the lack of effectiveness of NTX at low doses to reduce pain or the associate symptoms (Bested et al., 2023; Due Bruun et al., 2024; Ologunowa et al., 2025; Bruun et al., 2026). Those results agree with ours, where the use of antagonist of the opioid receptors induces hypersensitivity in the FM model, not the relief of the painful signs. Finally, the fact that availability of µ opioid receptors in encephalic structures related to pain processing in FM patients is reduced could also explain the failure of opioids to treat pain in FM patients (Harris et al., 2007).

We studied latent sensitization in ovariectomized animals since the lack of female gonadal hormones worsens allodynia (Hernandez-Leon et al., 2018). In addition, gonadal hormones influence the development and maintenance of allodynia in this model through the estrogen and progesterone receptors (Hernandez-Leon et al., 2018; De la Luz-Cuellar et al., 2019; 2023). Under our experimental conditions, very similar response to systemic naloxone was obtained in OVX and unoperated reserpinized animals, a total recall of the hypersensitivity. In addition, latent sensitization was unaffected by the surgery, which suggests that gonadal hormones have a poor influence on opioid compensatory mechanisms.

Despite the recent success in studying FM in animal models (Goebel et al., 2021; Caxaria et al., 2023), it is still necessary an affordable and convenient model in which studies of pharmacology and/or pathophysiology could be carried out with translational potential (Brum et al., 2022). The reserpine-induced model of pain seems to resemble another desirable characteristic of FM with the contribution of the present study, its chronic time-course.

In conclusion, our data suggests that reserpine-induced nociceptive hypersensitivity is a long-lasting painful condition masked by the compensatory release of β-endorphin and activation of spinal µ and δ receptors.

## Data Availability

The raw data supporting the conclusions of this article will be made available by the authors, without undue reservation.
