# Supplementary material for: Reserpine-induced fibromyalgia model in female rats is a long-lasting painful condition masked by antinociceptive endogenous opioids
Source: Front Pharmacol. 2026 Mar 18;17:1733691. doi: 10.3389/fphar.2026.1733691 (PMC13038963; doi:10.3389/fphar.2026.1733691)
Supplement: Supplementary file 1 [file Table1.docx]

Supplementary Table 1. ANOVA Results from the data analysis.

| Figure 1. |  |  |  |
| --- | --- | --- | --- |
| Thermonociceptive test |  |  |  |
| Two-way ANOVA results (Fig. 1B) | Treatment:  F (1, 10) = 38.61  P < 0.0001 | Time:  F (5.747, 57.47) = 8.296  P < 0.0001 | Interaction:  F (5.747, 57.47) = 6.891  P < 0.0001 |
| One-way ANOVA results (Fig. 1C) | Treatment:  F (5, 30) = 9.907  P < 0.0001 |  |  |
| Mechanonociceptive test |  |  |  |
| Two-way ANOVA results (Fig. 1D) | Treatment:  F (1, 10) = 235.8  P < 0.0001 | Time:  F (6.203, 62.03) = 6.217  P < 0.0001 | Interaction:  F (6.203, 62.03) = 5.787  P < 0.0001 |
| One-way ANOVA results (Fig. 1E) | Treatment:  F (5, 30) = 47.25  P < 0.0001 |  |  |
| Figure 2. |  |  |  |
| Thermonociceptive test |  |  |  |
| Two-way ANOVA results (Fig. 2A) | Treatment:  F (1, 10) = 4.365  P = 0.0632 | Time:  F (3.040, 30.40) = 45.35  P < 0.0001 | Interaction:  F (3.040, 30.40) = 3.121  P = 0.0398 |
| Unpaired t-test (Fig. 2B) | Treatment:  P = 0.1483 |  |  |
| Mechanonociceptive test |  |  |  |
| Two-way ANOVA results (2C) | Treatment:  F (1, 10) = 6.538  P = 0.0285 | Time:  F (5.081, 50.81) = 29.30  P < 0.0001 | Interaction:  F (5.081, 50.81) = 2.977  P = 0.0192 |
| Unpaired t-test (Fig. 2D) | Treatment:  P = 0.0017 |  |  |
| Figure 3. |  |  |  |
| Thermonociceptive test |  |  |  |
| Two-way ANOVA results (Fig. 3A) | Treatment:  F (2, 15) = 18.20  P < 0.0001 | Time:  F (4.708, 70.61) = 18.49  P < 0.0001 | Interaction:  F (9.415, 70.61) = 2.588  P = 0.0112 |
| One-way ANOVA results (Fig. 3B) | Treatment:  F (3, 20) = 15.45  P < 0.0001 |  |  |
| Mechanonociceptive test |  |  |  |
| Two-way ANOVA results (Fig. 3C) | Treatment:  F (3, 20) = 5.511  P = 0.0063 | Time:  F (6.348, 127.0) = 22.91  P < 0.0001 | Interaction:  F (19.04, 127.0) = 2.014  P = 0.0117 |
| One-way ANOVA results (Fig. 3D) | Treatment:  F (3, 20) = 7.993  P = 0.0011 |  |  |
| Figure 4. |  |  |  |
| Thermonociceptive test |  |  |  |
| Two-way ANOVA results (Fig. 4A) | Treatment:  F (1, 9) = 12.41  P = 0.0065 | Time:  F (3.605, 32.44) = 13.05  P < 0.0001 | Interaction:  F (3.605, 32.44) = 4.083  P = 0.0105 |
| Unpaired t-test (Fig. 4B) | Treatment:  P = 0.0082 |  |  |
| Mechanonociceptive test |  |  |  |
| Two-way ANOVA results (Fig. 4C) | Treatment:  F (3, 20) = 5.511  P = 0.0063 | Time:  F (6.348, 127.0) = 22.91  P < 0.0001 | Interaction:  F (19.04, 127.0) = 2.014  P = 0.0117 |
| Unpaired t-test (Fig. 4D) | Treatment:  P < 0.0001 |  |  |
